# High prevalence of *pfcrt*-CVIET haplotype in isolates from asymptomatic and symptomatic patients in south-central Oromia, Ethiopia

**DOI:** 10.1186/1475-2875-13-120

**Published:** 2014-03-27

**Authors:** Lemu Golassa, Nizar Enweji, Berhanu Erko, Abraham Aseffa, Göte Swedberg

**Affiliations:** 1 Aklilu Lemma Institute of Pathobiology, Addis Ababa University, Addis Ababa, Ethiopia; 2 Armauer Hansen Research Institute, Addis Ababa, Ethiopia; 3 Department of Medical Biochemistry and Microbiology, Uppsala University, Uppsala, Sweden

**Keywords:** *pfcrt*, Wild-type, Drug resistance, Chloroquine, *Plasmodium falciparum*, Mutations, Heterozygosity, Microsatellite, Ethiopia

## Abstract

**Background:**

As a result of extensive chloroquine resistance (CQR) in *Plasmodium falciparum* in late 1990s, Ethiopia replaced CQ with sulphadoxine-pyrimethamine (SP) as first-line drug, which in turn was replaced by artemisinin combination therapy in 2004. *Plasmodium falciparum* resistance to CQ is determined by the mutation at K76T of the *P. falciparum* chloroquine resistance transporter (*pfcrt*) gene. Understanding diversity in the *P. falciparum* genome is crucial since it has the potential to influence important phenotypes of the parasite such as drug resistance. Limited data is available regarding the type of *pfcrt* mutant allelic type, the effect of CQ withdrawal and diversity of the parasite population in south-central Oromia, Ethiopia.

**Methods:**

Finger-pricked blood spotted on Whatman 3MM filter papers were collected from falciparum malaria patients. Parasite DNA was extracted from individual blood spots on the filter papers. The presence of K76T mutations was determined using nested PCR for all isolates. Complete sequencing of mutations in *pfcrt* 72-76 was done for a set of randomly selected resistant isolates. Four microsatellite (MS) markers were analysed to determine the heterozygosity.

**Results:**

Although CQ was withdrawn for more than a decade, 100% of the parasites still carried the *pfcrt* K76T mutation. All isolates were mutant at the K76T polymorphism. Based on combinations of MS markers, seven different Ethiopian CQR variants (E1-E7) were identified. Heterozygosity (H_e_) for MS flanking the *pfcrt* chloroquine resistance allele ranged from 0.00 (mscrt -29, -29.268 kb) to 0.21 (mscrt -2, -2.814 kb). H_e_ ranged from 0.00 (msint 3, 0 kb) to 0.19 (msint 2, 0 kb) for MS within the *pfcrt* gene. Both intronic and MS flanking the *pfcrt* gene showed low levels of diversity.

**Conclusion:**

*pfcrt* CQR allele seems to be fixed in the study area. Of the different haplotypes associated with CQR, only the CVIET genotype was identified. No reversal to the wild-type has occurred in Ethiopia unlike in many Africa countries where CQR parasites declined after cessation of CQ use. Decreased diversity in CQR isolates surrounding *pfcrt* suggests CQ selection and homogenization among CQR parasite population. While mutation in msint 3 and mscrt -29 of the mutant *pfcrt* allele is being fixed, it seems that mutations in msint 2 and mscrt -2 are still evolving and may indicate the start of re-diversification of the population from a fixed 76 T population.

## Background

The emergence and spread of CQR *Plasmodium falciparum* in malaria-endemic countries has led to alterations in the anti-malarial treatment policy and the introduction of artemisinin-based combination therapies (ACT) for treatment of uncomplicated malaria [1]. The failure of CQ to clear *P. falciparum* asexual parasites in Ethiopia was first reported in 1986 in areas bordering Somalia, Kenya and Sudan. A steady increase in the number of refractory isolates was observed in all malarious areas in Ethiopia and as a result CQR *P. falciparum* became a major public health threat in 1990s [2]. In response to high levels of *P. falciparum* resistance to CQ and then sulphadoxine-pyrimethamine (SP), Ethiopia adopted artemether-lumefantrine (AL or Coartem©) as first-line therapy in 2004 [3-5]. According to the current national malaria diagnosis and treatment guidelines, AL and CQ are first-line treatment for uncomplicated *P. falciparum* and *Plasmodium vivax* infections, respectively. For all clinical infections without laboratory confirmation, AL, which is effective against both *P. falciparum* and *P. vivax*, is the first-line treatment. The reduced susceptibility of parasite strains to artemisinin necessitates assessment of the current status of CQR in Ethiopia. CQ is a drug of interest for study because of its efficacy, affordability, easy administration, prophylaxis potential, low toxicity and relatively few side effects [4].

It has been recognized that CQR falciparum malaria is caused by mutations in two genes, the *P. falciparum* CQ resistance transporter (*pfcrt*) and multidrug resistance transporter-1 (*pfmdr1*) however the former is a stronger predictor of CQR than *pfmdr1*. Polymorphisms in the *pfcrt* gene segregate precisely with two distinct drug response classes, considered either CQS or CQR. All *P. falciparum* clinical samples that are resistant to CQ contain the K76T mutation [6]. Amino acid change from lysine to threonine at position 76 (K76T) appears necessary for the resistance phenotype and is the most reliable molecular marker of resistance of the various *pfcrt* mutations. It is unfortunate to notice that strains of *P. falciparum* resistant to artemisinin have appeared in the Cambodia–Thailand border region [7]. While CQS strains are characterized by the CVMNK haplotype, irrespective of geographic origin, two major haplotypes defined by specific mutations at amino acid positions 72-76 of *pfcrt*, CVIET and SVMNT, are associated with the geographic origin of CQR [8]. The former haplotype is predominantly found in Southeast Asia and Africa, whereas the SVMNT haplotype is characteristic of South America, Papua New Guinea [9], and the Philippines [10].

A decrease in drug resistant *pfcrt* alleles has been reported following discontinuation of drug pressure [11] in Africa although this process can vary greatly between countries. A long-term decline in the use of CQ can lead to resurgence of drug-sensitive populations of *P. falciparum.* This raises the possibility that, in time, the drug could be re-introduced [12]. Understanding the influence of CQ withdrawal on local malaria parasite populations has great significance. In Malawi, for instance, the frequency of the mutation declined from 85% in 1992 to 13% in 2000 in one study [13] and from 17% in 1998 to 2% in 2000 in another study [14] after CQ was withdrawn as the first- line drug. Studies in Kenya [15] and China [16] have reported complete and partial recovery of CQS parasites after the use of CQ was abandoned. This recovery was thought to be due to the re-introduction of susceptible parasites harbouring a CQS *pfcrt*[17,18], although the possibilities of back mutation in *pfcrt* at position 76 may not be underestimated. Continued surveillance of markers of resistance to withdrawn drugs has been indicated with the prospect that decreases in resistance may make it possible to reuse this safe and cheap drug [4,17]. The overall trend in the reversal of the resistance could be related to a country’s drug policies although data regarding the impact of CQ withdrawal on circulating parasite population is not well studied in Ethiopia.

When advantageous mutations such as drug resistance mutations spread through a population, unrelated markers that flank these loci are carried along through linkage disequilibrium by a “hitch-hiking effect”, thereby removing genetic variation from chromosomal regions surrounding the locus [19]. Unlike in CQS alleles, there is a marked drop in variability and an increase in linkage disequilibrium with CQR associated alleles in ~40 kb region flanking the *pfcrt* gene [8]. MS repeats, occurring on the average approximately every 1 kb [20,21], are common in the *P. falciparum* genome and have been recently used to analyse both intra- and inter-population relationships among drug-resistant *P. falciparum* strains.

Drug treatment has not only resulted in positive selection at codons conferring resistance but also alleles at loci closely linked to these genes, due to limited recombination in the chromosome regions containing these genes since drug selection was imposed [22]. If a particular mutation in the target molecule is fixed in the parasite population due to the drug pressure, its flanking MS markers will start showing minimum variation [23]. Thus, selective sweeps for drug-resistant genotypes may have restricted the genetic diversity of this parasite [8]. MS markers associated with drug resistance enabled identification of drug resistance origin as well as tracing spread of these mutations [24]. Drug- resistant genotypes are characterized by reduced diversity around the major resistant alleles [25]. Thus, the expected heterozygosity (H_e_) values of the flanking MSs will be reduced among mutant parasites. Diversity of haplotypes existed in the parasite population before CQR but after the selection, parasites bearing the *pfcrt*76K allele are eliminated and those with *pfcrt*76T survive and expand in the population. Thus, in areas where CQ has been widely used, there will be homogeneity of the loci in this chromosome region of the resistant parasites around the *pfcrt* whereas diversity of MS alleles will still be found in sensitive parasites. Despite high level of CQR in Ethiopia, pertinent data regarding the type of mutant *pfcrt* genotype are lacking.

Applications of MS markers flanking the genes have revealed that drug resistance appears to have resulted from the migration of limited resistant lineages to many endemic regions however the resistance haplotypes present in Ethiopia is not known. Despite the migration of resistant lineages, multiple resistant lineages independently evolved in several endemic regions. Indeed, the geographic origin and spread of resistance can be estimated by the genotyping of *pfcrt* at positions 72-76 and MS haplotyping flanking this locus. Although Ethiopia is highly endemic for *P. falciparum* malaria and CQR *P. falciparum* isolates occur widely, there is scarcity of data regarding the *pfcrt* genotypes and diversity among the parasite population. Authors hypothesize that although CQ was replaced by ACT for treatment of *P. falciparum* malaria in the country, unlike in many African countries, the withdrawal CQ may not result in the reduction of CQR genotype for the fact that it is still the first-line treatment for *P. vivax* and co-infections are inevitable. The purpose of this study was, therefore, to determine the CQR haplotypes, to unveil the magnitude of *P. falciparum* CQR genotypes 14 years after its withdrawal and to determine the genetic diversity in CQR alleles in south-central Oromia, Ethiopia.

## Methods

### Study area and population

The study was conducted in Shalla District (07 17′ 34.2 S, 038′ 21″’ 46.3 W), West Arsi Zone, Oromia, located 251 km from, Addis Ababa, Ethiopia. The district is 1500-2300 m above sea level. It has a land-mass of about of 5,932 sq km with a projected population of 176,671 living in malarious areas. The area is characterized by relatively high rates of poverty. The inhabitants are mainly supported by rain-dependent cultivation of maize, tef (*Eragrostis tef*), wheat and sorghum. In the study area, malaria is caused by two dominant species, *P. falciparum* and *P.vivax*. The area experiences distinct wet and dry seasons. Malaria transmission is seasonal and unstable in the study area.

### Collection of *Plasmodium falciparum* isolates

A total of 99 *P. falciparum* isolates were collected in Shalla District from November through December 2012. Detailed procedures for parasite collection were previously described [26]. Forty-nine isolates were collected from febrile (clinical/symptomatic) patients and 50 from asymptomatic subjects during community-based screening of asymptomatic *P. falciparum* carriers. Malaria diagnosis was done by rapid diagnostic test (RDT) (SD BIOLINE Malaria Ag P.f/P.v POCT test kits, Standard Diagnostic, Inc, Germany, LOT No:145021) under field conditions and microscopy at Adama/Nazareth Malaria Control Centre. Finger-prick blood samples were spotted on Whatman 3MM filter papers for parasite DNA extraction.

### DNA extraction and RFLP-PCR

Parasites DNA were extracted from blood spotted on Whatman 3MM filter papers using the chelex extraction method [27]. DNA was prepared from 3-mm discs removed from each blood spot using a sterile hole-punch. The cut section of the spot was soaked in a 1 ml, 0.5% saponion-1x phosphate buffered saline (PBS) overnight in a 96-deep-well plate. The cut section of filter paper was then washed twice in 1xPBS, and was then boiled for 10 min in 100 μl PCR quality water with 50 μl 20% chelex suspension (pH 9.5). DNA was amplified by nested PCR (LifePro thermal cycler Bioer). The restriction fragment length polymorphism (RFLP) protocol was used to identify the K76T mutation, essentially as described previously [6]. For restriction enzyme digestions, PCR products were used without purification. Five μl of the nested-PCR products were digested with one unit of *Apo*I restriction enzyme at 55°C for three hours, which yielded two fragments (100 bp and 45 bp) on 2% agarose gel in the case of wild-type (K76), but it remained uncut (hence one band) when the 76 T mutation was present. Both PCR products and digestion fragments were visualized by ethidium bromide under UV light.

### *pfcrt* genotyping and sequencing

Determination of polymorphisms at *pfcrt* was performed by PCR-RFLP method. The *pfcrt* gene was amplified by nested PCR using two sets of primers designed to amplify a region of exon 2 including known polymorphic sites at amino acid positions 72-76. Primer sequences and PCR conditions were as shown elsewhere [28]. *Apo I*, which cuts the K76 codon, was used to distinguish wild-type from the mutant. After screening the PCR products on agarose gel to identify the resistant and the wild type, the primary products of nested PCR-RFLP resistant to the *Apo*I (Fermentas) digestion, showing only one band (mutant genotypes), were subjected to DNA sequencing. Samples were purified before sequencing using GeneJET PCR purification kit (Thermo Scientific, Lithuania) and sequenced using an automatic sequencer ABI3730 at Uppsala Genome Centre (Uppsala University, Sweden).

### Microsatellite analysis

Four highly polymorphic MS loci (msint 2, msint 3, mscrt -2 and mscrt -29) were used for determining variation among *pfcrt* mutant parasites. Two of these markers (msint 2 and msint 3) were located in introns and the other two (mscrt -2 and mscrt -29) were flanking loci situated downstream of the *pfcrt* gene [8,20,21]. MS variations in the number of repeats in *pfcrt* were measured using a PCR protocol previously described [29]. Briefly, each MS marker was amplified by semi-nested PCR in a LifePro thermal cycler (Bioer) in 20 μl reaction containing 2 μl of genomic DNA template, 0.5 unit of *Taq* polymerase, 2.5 μl of 10× reaction buffer, and 0.4 mM of each dNTP. Cycling parameters were set for each primer pairs. Length variation of labelled PCR products was measured on an ABI PRISM 3730XL DNA Analyzer (Applied Biosystems, Sweden). The GENESCAN and GENOTYPER software (Applied Biosystems) were used to measure allele length and to quantify peak heights. Haplotypes were classified as different if they contained ≥ one different alleles across all loci. Samples presenting multiple alleles at any of the four loci were considered mixed infections and were not used for multilocus MS haplotype construction and other analyses. Nevertheless, it is possible that nonspecific marker binding will cause noise in the form of minor peak heights during the capillary electrophoresis reaction. As a result, the standard method is to exclude minor peaks less than one third the height of the major peaks (alleles) [30]. MS haplotypes were determined for all *pfcrt* mutant isolates. During every MS reaction, a Dd2 reference isolate was included as a positive control while water was used as negative control.

### Ethical clearance

Study protocols were reviewed and approved by Institutional Review Boards of Aklilu Lemma Institute of Pathobiology, Addis Ababa University and of the Armauer Hansen Research Institute as well as the National Research Ethics Review Committee. Ahead of enrolment, written informed consent was obtained from all study subjects. In the case of children, consent was obtained from a parent or legal guardian. Illiterate people signed with their fingerprint.

### Statistical analysis

The genetic variation in terms of expected heterozygosity (H_e_) for each MS locus was calculated using the following formula, H_e_ = [n/ (n-1)] [1-ΣPi^2^] where *n* is the number of alleles in the sample and *p* is the frequency of the *i*^th^ allele. When two or more polymorphisms were detected, the isolates were considered to be mixed infections and thus excluded from the analysis. H_e_ values ranges from 0 to 1 where 0 means that there is no difference between individual isolates and 1 means that all individual isolates are unique and population groups are distinct from each other.

## Results

### Prevalence of molecular markers for *pfcrt*

Results from this study provide important information about the effect of CQ usage on the population structure of Ethiopian *P. falciparum* parasites. In this study, *pfcrt* K76T mutation involved in CQR analysed in 99 *P. falciparum* samples (forty-nine from clinical patients and 50 from asymptomatic subjects) collected 14 years after withdrawal of CQ for the treatment of uncomplicated *P. falciparum* from the study areas. Analysis of samples showed that 100% (n = 99) of the patients harboured the pure mutant allele of *pfcrt* 76 T. Twenty of the fragments that were resistant to *Apo*I digestion (76 T genotype) were randomly selected for complete sequence for amino acids positions 72-76. All the isolates harboured the *pfcrt*-CVIET genotype. No single wild-type, CVMNK *pfcrt* genotype, was detected in the study area.

### Mapping of genetic relatedness of resistance mutants

To analyse the genetic relatedness of the alleles carrying the CVIET haplotype, intronic (msint 2, msint 3) and flanking (mscrt -2, mscrt -29) MS loci were analysed for all the isolates included in this study. Of 99 isolates harbouring *pfcrt* mutant genotypes, 79 samples were successfully analysed for MS haplotypes. Using four MS, there were 79 single-clone infections. In eight of the isolates MS were not successfully amplified and excluded from analysis. Twelve samples were also not included in calculating allelic frequencies because they had either multiple alleles (>one peak in the sequencing electropherogram) or missing data at one or more codons. 85.7% (10/12) of the samples presenting multiple alleles and hence excluded from analysis were those collected from asymptomatic individuals. Thus, MS analysis was done for 79 mutant isolates. There were seven unique MS haplotypes detected in the 79 *P. falciparum* isolates. To assess the genetic relatedness of *pfcrt* mutants, a size-based haplotype variations at all MS markers was constructed. Haplotype was defined as the specific combination of the alleles of the following four MS markers: msint 2 (0 kb), msint 3 (0 kb), mscrt -2 (-2.814 kb) and mscrt -29 (-29.268 kb). Only one intronic and one flanking marker showed more than one allele (Table 1). The number of alleles per locus ranged from one (at msint 3 and mscrt -29) to four (msint 2). Heterozygosity values were 0.19 and 0.21 for msint 2 and mscrt -2, respectively (Table 2). Reduced variation in markers spanning a -29 kb region around *pfcrt* was observed in the study area.

**Table 1:** ***pfcrt *** **allelic types defined by mutations in positions amino acids 72-76 and polymorphisms in MS markers (intronic and flanking) in 79 *P. falciparum *** **isolates in Shalla district, south-central Oromia, Ethiopia**

**Haplotype ID**	**MS distance from** ***pfcrt***				***pfcrt*** **genotype/a.a at** ***pfcrt*** **76**	**Isolate sources**		**No. of isolates, (n = 79)**	**Prevalence**
	**mscrt -29 (-29.268 kb)**	**mscrt -2 (-2.814 kb)**	**msint 3 (0 kb)**	**msint 2 (0 kb)**	**CVIET**	**Clinical**	**Asymptomatic**		
E1	149	179	198	204	CVIET	35	30	65	82.3%
E2	149	181	198	204	CVIET	3	2	5	6.3%
E3	149	179	198	209	CVIET	0	2	2	2.5%
E4	149	179	198	215	CVIET	1	1	2	2.5%
E5	149	179	198	197	CVIET	0	1	1	1.3%
E6	149	181	198	215	CVIET	3	0	3	3.8%
E7	149	177	198	204	CVIET	1	0	1	1.3%

Haplotypes ID (E1-E7) were classified as different if they contained ≥ one different alleles across all loci. Allelic size at each MS locus is shown.

**Table 2:** **Number of alleles and expected heterozygosity per MS locus for *pfcrt *** **on chromosome 7 (n = 79)**

**Locus**	**MS position with respect to** ***pfcrt***^**a**^	**No. of alleles per MS loci**	**H**_**e**_**Mean (±s. d)**
Msint 2	0 kb	4	0.19 (0.05)
Msint 3	0 kb	1	0.00 (0.00)
Mscrt -2	-2.814 kb	3	0.21 (0.05)
Mscrt -29	-29.268 kb	1	0.00 (0.00)

^a^Upstream distances were calculated from the gene’s start codon and downstream distances were calculated from the gene’s stop codon.

### Microsatellite analysis of isolates from symptomatic and asymptomatic subjects

The 79 isolates with CVIET belonged to seven closely related haplotypes. Alleles were determined as differences in repeat length. There were seven different Ethiopian CQR haplotypes (E1-E7) identified in this study. The most prevalent haplotype, E1, (n = 65) had allelic combination of 204-198-179-149 at the MS loci of 0 kb, 0 kb, -2.814 kb and -29.268 kb, respectively (Table 1). The other haplotypes, E2 (n = 5), E3 (n = 2), E4 (n = 1), E5 (n = 1), E6 (n = 3) and E7 (n = 2) and their respective allelic combinations is found in Table 1. Three allelic combinations in E1, E2 and E4 were shared among all the mutant isolates at the MS loci of msint 2, msint 3, mscrt -2 and mscrt -29, respectively. Two allelic combinations in E6 and E7 were specific to isolates collected from clinical patients. Two other allelic combinations in E3 and E5 were found only in isolates collected from asymptomatic subjects. Strong CQ selection together with low local recombination rates (low intensity of transmission) in the study area may account for reduced variation at *pfcrt.* Allelic sizes of the four MS with respect to all the isolates are found in Additional file 1.

## Discussion

The widespread resistance to CQ and other-antimalarials occurring in Ethiopia has fostered the deployment of ACT for falciparum malaria. ACT (AL/Coartem©), has been the choice of *P. falciparum* therapy in Ethiopia since 2004. At the time of CQ withdrawal, the prevalence of the *pfcrt*-76 T resistance marker was estimated to have been 100% [31] in Ethiopia. Meanwhile, declining resistance to CQ observed in *P. falciparum* in many malarious regions after its withdrawal suggests a possibility of future reintroduction of the drug.

In this study the prevalence of K76T mutation was 100% although CQ has been withdrawn since 1999 in Ethiopia. The fact that all isolates were mutant mean that reversal from CQR to wild-type has not occurred and the parasites seem under continuous selection pressure of CQ. The high level of *pfcrt* K76T mutation observed in this study is in agreement with a study done in a rural hospital in southern Ethiopia [32]. For various reasons, drug resistance is expected to drop after the removal of selective pressure [33] although CQR has remained high in the study site (Ethiopia) which is in concordance with a study conducted in Yunnan Province, China [33]. It is highly likely that the use of CQ as a first-line treatment for *P. vivax*, which accounts for approximately 40% of all malaria cases, may have contributed to the persistence of the K76T mutation in Ethiopia. The use of CQ for the treatment of vivax malaria in areas with CQR *P. falciparum* could increase the transmission of falciparum malaria and the selection of mutant strains. In areas where *P. falciparum* and *P.vivax* co-exist, mixed-species infections are common and undoubtedly, CQ selective pressure on *P. falciparum* is retained in such areas. Nonetheless, some of these co-infections are often diagnosed as vivax malaria by conventional microscopic examination and treated with CQ, when cryptic CQR *P. falciparum* infections emerge after treatment [34]. For instance, using PCR, *P. falciparum* was identified in 18.7% (3/16) and 35.3% (6/14) of RDT-and microscopy-confirmed *P. vivax* infections, respectively in the study area (unpublished). The persistent use of CQ for the treatment of *P. vivax* is, therefore, responsible for the absence of decline of *P. falciparum* CQR in Ethiopia.

This is the first report in Ethiopia, to the best of author’s knowledge, to uncover the presence of a single CQR mutant *pfcrt* genotype, CVIET, the most prevalent haplotype in Africa. In this study, the SVMNT mutant haplotype was not identified. Studies have shown that the withdrawal of CQ in Kenya [15], Malawi [13], China [35] and Tanzania [36], have resulted in the reversion to the ancestral state of CQS from the CVIET haplotype. In contrast, no change in the prevalence of the mutant *pfcrt* allele occurred in Gabon [37,38] which changed its national treatment guidelines from CQ to the combination of artesunate plus aminoquinoline. But in Ghana [39] which also changed its national treatment guidelines from CQ to ACT (the combination of artesunate plus aminoquinoline), a decrease in the prevalence of the mutant *pfcrt* allele was observed in northern parts of the country. Indeed, it is hardly possible to arrive at a definitive conclusion as to whether the absence of CQ in the field or the use of ACT has accounted for the re-emergence of wild types for *pfcrt*[13,40]. Kamugisha *et al*., [41] attributed the observed differences in the decline of CQR in Mwanza, Tanzania and Iganga, Uganda to country-specific drug change policies. Since the emergence of a new haplotype can be spontaneous, continuous monitoring is necessary as the SVMNT haplotype initially absent from Africa suddenly appeared in Tanzania in 2004 [42].

Studies have shown that CQS parasites (*pfcrt*76K) exhibited extensive diversity at all loci while CQR strains (*pfcrt*76T) showed reduced diversity around the *pfcrt* gene but high diversity for loci on other chromosomes [39-42]. In this study, the CQR isolates showed reduced diversity with respect to all MS markers (two introns and two flanking). The isolates were 89.9, 100, 88.6 and 100% identical with respect to msint 2 (0 kb), msint 3 (0 kb), mscrt -2 (-2.814 kb) and mscrt -29 (-29.268 kb), respectively. Although some allelic combinations were shared among parasites collected from asymptomatic subjects and clinical samples, there are certain allelic combinations specific to each repertoire isolate types. Of the seven haplotypes identified in the study area, haplotye E1 is the most prevalent allelic type. But whether these allelic combinations could be linked to either symptomatic or asymptomatic infections needs further research. It can be inferred from this study that parasites identified from asymptomatic subjects are more diverse than those present in symptomatic infections (clinical) sample sets. Despite low malaria transmission setting in the study area, asymptomatic parasite carriage is still associated with a higher multiplicity of infection. The number of MS alleles varied among the *pfcrt* introns and flanking loci successfully analyzed, with msint 2 displaying the greatest number of alleles (n = 4). On the other hand, msint 3 and mscrt -29, showed no polymorphisms. In this study, the CVIET mutant genotype showed variation in 11.1% (msint 2) and 13.4% (mscrt -2), but the isolates were 100% identical with respect to msint 3 and mscrt -29 allelic sizes. In *P. falciparum* strains, very low or no heterozygosity would be expected for msint 2 and msint 3 [8]. Diversity observed in msint 2 in this study is supported by a study in a Papua New Guinea [43] that has indicated the continuing evolution of the region’s parasite populations. Given that msint 2 and msint 3 are similar in their length and nucleotide content, it is unclear why the mutant strains show variability in msint 2 but not in msint 3. The possible explanation for the observed reduction in MS allelic diversity in the closest proximity to the *pfcrt* allele is the result of strong CQ selection pressure. Because CQ was responsible for homogenizing genetic diversity at polymorphic sites in close physical linkage with CQR-associated alleles [8].

Genetic diversity of the malaria parasite at a given region is correlated with the local transmission intensity. It has been indicated that drug selection tends to diminish genetic diversity, especially near the resistance-determinant locus [8]. The low heterozygocity of intronic and flanking MS alleles observed in this study may indicate that parasite population in the study area is genetically homogeneous, which could be the result of the CQ-selective sweep. Although analysis of MS loci around the *pfcrt* gene provides suggestive evidence for selection around this gene, the absence of parasites bearing sensitive *pfcrt* (CVMNK haplotype) alleles in this population limits the strength of this conclusion. Hence, it was difficult to compare patterns of MS variation in both sensitive and resistant *pfcrt* alleles in this study. The other limitation of this study was the fact that other mutations at *pfcrt* codons associated with CQR were not examined also absence of mscrt -29 variations limits the conclusive power.

## Conclusions

The present findings revealed the presence of only one *pfcrt* mutant genotype, CVIET, in this study area. The continued use of CQ for the treatment of *P. vivax* could be one major reason for the persistence of mutant the *pfcrt* allele in the study area. Given the incomplete withdrawal of CQ in Ethiopia, no reversal to the wild-type was observed although recent reports have shown that removal of CQ pressure in Africa is associated with a return of the wild-type *pfcrt* allele carrying strains and CQ sensitivity. Since H_e_ was low both at intronic and flanking MS loci, the isolates were more or less homogenous. Indeed, homogeneity is expected in resistant parasites in areas where CQ has been widely used. The genetic structure determined by introns- and *pfcrt*-flanking markers indicates at least seven subpopulations of Ethiopian *P. falciparum* isolates, circulating in the study area. Further studies on the prevalence markers and genetic diversity within parasite populations are required in Ethiopia since the conclusions of the present study are limited to samples collected in the study area.

## Competing interests

The authors declare that they have no competing interests.

## Authors' contributions

LG, BE, AA and GS designed and were involved in all stages of this study. LG did the sample collection and prepared the draft manuscript. BE and AA coordinated the field work. LG, NE and GS performed molecular analysis and MS genotyping. All authors contributed to interpretation of data, writing and revising the manuscript and have approved the final version.

## Supplementary Material

Additional file 1 **Microsatellite haploptyes of chloroquine-resistant *Plasmodium falciparum *** **parasites in the region of chromosome 7 surrounding the gene encoding the *P. falciparum *** **chloroquine resistance transporter (*pfcrt*) among Ethiopian *P. falciparum *** **isolates.** Click here for file
