# Supplementary material for: High prevalence of pfcrt-CVIET haplotype in isolates from asymptomatic and symptomatic patients in south-central Oromia, Ethiopia
Source: Malar J. 2014 Mar 27;13:120. doi: 10.1186/1475-2875-13-120 (PMC3986696; doi:10.1186/1475-2875-13-120)
Supplement: Additional file 1 — Microsatellite haploptyes of chloroquine-resistant Plasmodium falciparum parasites in the region of chromosome 7 surrounding the gene encoding the P. falciparum chloroquine resistance transporter ( pfcrt ) among Ethiopian P. falciparum isolates. [file 1475-2875-13-120-S1.doc]

**Additional File 1. Microsatellite haploptyes of chloroquine-resistant *Plasmodium falciparum* parasites in the region of chromosome 7 surrounding the gene encoding the *P. falciparum* chloroquine resistance transporter (*pfcrt*) among Ethiopian *P. falciparum* isolates.**

| **Distance of microsatellite loci from *pfcrt*** | | | | ***pfcrt* genotype/**  **amino acid at *pfcrt* 76** |
| --- | --- | --- | --- | --- |
| **mscrt 29**  **(-29. 268kb)** | **mscrt 2**  **(-2. 814kb)** | **msint 3**  **(0kb)** | **msint 2**  **(0kb)** |  |
| 149 | 179 | 198 | 204 | CVIET |
| 149 | 179 | 198 | 204 | CVIET |
| 149 | 179 | 198 | 204 | CVIET |
| 149 | 179 | 198 | 204 | CVIET |
| 149 | 179 | 198 | 204 | CVIET |
| 149 | 179 | 198 | 204 | CVIET |
| 149 | 179 | 198 | 204 | CVIET |
| 149 | 179 | 198 | 204 | CVIET |
| 149 | 179 | 198 | 204 | CVIET |
| 149 | 179 | 198 | 204 | CVIET |
| 149 | 179 | 198 | 204 | CVIET |
| 149 | 179 | 198 | 204 | CVIET |
| 149 | 179 | 198 | 204 | CVIET |
| 149 | 179 | 198 | 204 | CVIET |
| 149 | 179 | 198 | 204 | CVIET |
| 149 | 179 | 198 | 204 | CVIET |
| 149 | 179 | 198 | 204 | CVIET |
| 149 | 179 | 198 | 204 | CVIET |
| 149 | 179 | 198 | 204 | CVIET |
| 149 | 179 | 198 | 204 | CVIET |
| 149 | 179 | 198 | 204 | CVIET |
| 149 | 179 | 198 | 204 | CVIET |
| 149 | 179 | 198 | 204 | CVIET |
| 149 | 179 | 198 | 204 | CVIET |
| 149 | 179 | 198 | 204 | CVIET |
| 149 | 179 | 198 | 204 | CVIET |
| 149 | 179 | 198 | 204 | CVIET |
| 149 | 179 | 198 | 204 | CVIET |
| 149 | 179 | 198 | 204 | CVIET |
| 149 | 179 | 198 | 204 | CVIET |
| 149 | 179 | 198 | 204 | CVIET |
| 149 | 179 | 198 | 204 | CVIET |
| 149 | 179 | 198 | 204 | CVIET |
| 149 | 179 | 198 | 204 | CVIET |
| 149 | 179 | 198 | 204 | CVIET |
| 149 | 179 | 198 | 204 | CVIET |
| 149 | 179 | 198 | 204 | CVIET |
| 149 | 179 | 198 | 204 | CVIET |
| 149 | 179 | 198 | 204 | CVIET |
| 149 | 179 | 198 | 204 | CVIET |
| 149 | 179 | 198 | 204 | CVIET |
| 149 | 179 | 198 | 204 | CVIET |
| 149 | 179 | 198 | 204 | CVIET |
| 149 | 179 | 198 | 204 | CVIET |
| 149 | 179 | 198 | 204 | CVIET |
| 149 | 179 | 198 | 204 | CVIET |
| 149 | 179 | 198 | 204 | CVIET |
| 149 | 179 | 198 | 204 | CVIET |
| 149 | 179 | 198 | 204 | CVIET |
| 149 | 179 | 198 | 204 | CVIET |
| 149 | 179 | 198 | 204 | CVIET |
| 149 | 179 | 198 | 204 | CVIET |
| 149 | 179 | 198 | 204 | CVIET |
| 149 | 179 | 198 | 204 | CVIET |
| 149 | 179 | 198 | 204 | CVIET |
| 149 | 179 | 198 | 204 | CVIET |
| 149 | 179 | 198 | 204 | CVIET |
| 149 | 179 | 198 | 204 | CVIET |
| 149 | 179 | 198 | 204 | CVIET |
| 149 | 179 | 198 | 204 | CVIET |
| 149 | 179 | 198 | 204 | CVIET |
| 149 | 179 | 198 | 204 | CVIET |
| 149 | 179 | 198 | 204 | CVIET |
| 149 | 179 | 198 | 204 | CVIET |
| 149 | 179 | 198 | 204 | CVIET |
| 149 | 181 | 198 | 204 | CVIET |
| 149 | 181 | 198 | 204 | CVIET |
| 149 | 181 | 198 | 204 | CVIET |
| 149 | 181 | 198 | 204 | CVIET |
| 149 | 181 | 198 | 204 | CVIET |
| 149 | 179 | 198 | 209 | CVIET |
| 149 | 179 | 198 | 209 | CVIET |
| 149 | 179 | 198 | 215 | CVIET |
| 149 | 179 | 198 | 215 | CVIET |
| 149 | 179 | 198 | 197 | CVIET |
| 149 | 181 | 198 | 215 | **CVIET** |
| 149 | 181 | 198 | 215 | CVIET |
| 149 | 181 | 198 | 215 | CVIET |
| 149 | 177 | 198 | 204 | CVIET |

MS alleles in the least frequent haplotype are shown in white.
